# Characterization and site-directed mutagenesis of Wzb, an O-phosphatase from *Lactobacillus rhamnosus*

**DOI:** 10.1186/1471-2091-9-10

**Published:** 2008-04-03

**Authors:** Gisèle LaPointe, Danièle Atlan, Christophe Gilbert

**Affiliations:** 1 STELA Dairy Research Centre, INAF, Université Laval, Québec, QC, G1V 0A6, Canada; 2 Université de Lyon, Lyon, F-69003, France; Université Lyon 1, Lyon, F-69003, France; INSA de Lyon, Villeurbanne, F-69621, France; BayerCropScience, Lyon, F-69263, France; CNRS, UMR5240, Unité microbiologie, adaptation et pathogénie, Villeurbanne, F-69622, France

## Abstract

**Background:**

Reversible phosphorylation events within a polymerisation complex have been proposed to modulate capsular polysaccharide synthesis in *Streptococcus pneumoniae*. Similar phosphatase and kinase genes are present in the exopolysaccharide (EPS) biosynthesis loci of numerous lactic acid bacteria genomes.

**Results:**

The protein sequence deduced from the *wzb *gene in *Lactobacillus rhamnosus *ATCC 9595 reveals four motifs of the polymerase and histidinol phosphatase (PHP) superfamily of prokaryotic O-phosphatases. Native and modified His-tag fusion Wzb proteins were purified from *Escherichia coli *cultures. Extracts showed phosphatase activity towards tyrosine-containing peptides. The purified fusion protein Wzb was active on *p*-nitrophenyl-phosphate (*p*NPP), with an optimal activity in presence of bovine serum albumin (BSA 1%) at pH 7.3 and a temperature of 75°C. At 50°C, residual activity decreased to 10 %. Copper ions were essential for phosphatase activity, which was significantly increased by addition of cobalt. Mutated fusion Wzb proteins exhibited reduced phosphatase activity on *p*-nitrophenyl-phosphate. However, one variant (C6S) showed close to 20% increase in phosphatase activity.

**Conclusion:**

These characteristics reveal significant differences with the manganese-dependent CpsB protein tyrosine phosphatase described for *Streptococcus pneumoniae *as well as with the polysaccharide-related phosphatases of Gram negative bacteria.

## Background

The importance of protein phosphorylation in signal transduction has been amply demonstrated for the regulation of both eukaryotic and prokaryotic cellular processes (reviewed in [1-3]). Protein phosphorylation on serine, threonine or tyrosine residues is catalyzed by protein kinases while dephosphorylation is catalyzed by protein phosphatases. The activity of these enzymes can be modulated by external stimuli, or by regulatory or targeting subunits.

Prokaryotic phosphatases can be classified into four superfamilies, based on amino acid sequence comparison [1-4]. The phosphoprotein phosphatase (PPP) family contains mainly eukaryotic members, and is not widespread in bacterial genomes. The catalytic domain contains two metal ions (Mn^2+^, Fe^2+ ^or Fe^3+^) in the active site. Among the eukaryotic members, the catalytic domain is conserved, but outside this region, sequence diversity is higher. The substrate specificity and functions of the eukaryotic PPP proteins are modulated by regulatory and targeting subunits. However, such interactions with other subunits have not yet been shown for prokaryotic PPP proteins. The second superfamily consists of the Mg^2+ ^or Mn^2+^-dependent protein phosphatases (PPM). The divalent cations are bound by four conserved aspartic residues, and the catalytic mechanism resembles that of PPP family protein phosphatases. In addition to the catalytic domain, proteins in the PPM family can contain other domains with various functions, such as membrane-spanning domains, protein-protein interaction domains or sensor domains.

The third superfamily contains polymerases such as DNA polymerase III α subunits and X-family DNA polymerases as well as histidinol phosphatases (PHP) from yeast and bacteria such as *Lactococcus lactis*. Although very diverse in function, many members are not protein phosphatases, but are proposed to hydrolyze inorganic pyrophosphate in order to facilitate the continuation of polymerisation. Histidinol phosphatase removes the phosphate group from histidine during the biosynthesis of this amino acid in yeast. CpsB from *Streptococcus pneumoniae *is a member of the PHP superfamily, and has been shown to be a protein phosphatase that is Mn^2+^-dependent [5]. Members of the PHP superfamily are also found in other Gram positive species such as *Bacillus subtilis*, where PtpZ can dephosphorylate its cognate tyrosine kinase PtkA, as well as UDP-glucose dehydrogenases (Ugd or TuaD), thus suggesting a role in teichuronic acid synthesis [6-8]. All members of this superfamily share four consensus motifs containing histidine and aspartate residues that are proposed to carry out the metal-dependent hydrolysis of phosphoester bonds. Tyrosine kinases coded by genes adjacent to PHP type phosphatase genes have been shown to be substrates for PHP activity [6].

Finally, the fourth PTP superfamily of phosphotyrosine protein phosphatases actually contains two distinct subfamilies sharing a common catalytic mechanism, but that result from convergent evolution [9]. The members of the first subgroup have dual specificity; serine/threonine and tyrosine, while the members of the second subgroup are the low molecular weight PTPs specific for tyrosine residues. PTP-type enzymes have been identified by genome search and analysis in a number of bacterial species [10-12]. During the two-step phosphatase reaction, a phosphocysteine intermediate is generated through transfer of the phosphate to the cysteine of the CX_5_R motif. Mutation of this cysteine in the motif of the SptP protein from *Salmonella typhimurium *eliminates hydrolysis of phosphotyrosine substrates [13]. The arginine promotes substrate binding by forming salt bridges with the phosphoryl group [14] and also stabilizes the phosphoenzyme intermediate [15]. Two LMW-PTPs have been identified and characterized from *Bacillus subtilis*, and were shown to be involved in resistance to ethanol stress [16]. In Gram negative bacteria, LMW-PTPs have roles in regulating capsule composition [17] as well as modulating the phosphorylation state of transcription factors in the heat shock response [18]. Tyrosine phosphorylation, in particular, has also been shown to have a role in pathogenicity through the control of exopolysaccharide production [19].

Emerging roles for bacterial tyrosine phosphorylation systems are diverse, ranging from adaptation, virulence and stress responses to DNA metabolism, and cell division as well as motility and sporulation [6]. Reversible phosphorylation events within a polymerisation complex have been proposed to modulate capsular polysaccharide synthesis in streptococci [20-22]. Extracellular polysaccharide production in any form (secreted, attached or capsular) is an example of a metabolic activity that consumes energy and carbon in a product that is not readily accessible as a nutrient source. The control of polymer production can be exerted at the transcription level, or post-translationally. Polymerisation must be strictly controlled with respect to precursor availability and the energy necessary to form glycosidic bonds as well as to transport the units out of the cell. Bender and Yother [20] have proposed that a stable complex consisting of CpsB, CpsC, CpsD and ATP enhances capsule synthesis. Similar systems have been proposed for EPS synthesis by *L. lactis *[23] and *Streptococcus thermophilus *[24]. They showed that CpsB can dephosphorylate and inhibit the phosphorylation of CpsD, while CpsD is a tyrosine kinase that requires the presence of the accessory transmembrane protein, CpsC, for intra- and inter-molecular phosphorylation. Deletion mutants of *cps2C *and *cps2D *or *epsC *and *epsD *do not produce any capsular material or EPS [20]. In addition, Morona et al. [25] showed that two simultaneous point mutations (D199N and H201Q) in one conserved motif of CpsB result in loss of the capsule, equivalent to the phenotype of the mutant in which the entire *cpsB *gene was deleted. Nothing is known of the importance of other conserved residues for the function of phosphatases of this type.

There are important differences between Gram negative and Gram positive bacteria with respect to the polysaccharide polymerization mechanism. In *E. coli*, the tyrosine kinase-phosphatase pair Wzc-Wzb has been shown to participate in colanic acid synthesis [26]. Wzc was shown to autophosphorylate on tyrosine, and is dephosphorylated by Wzb [17]. A similar mechanism has been proposed for *S. pneumoniae *(CpsB dephosphorylates CpsD; Morona 2003), as well as for *Streptococcus thermophilus *[24] and *Lactococcus lactis *[23]. However, there are two major differences between these Gram negative and Gram positive systems. Wzc has an N-terminus with two transmembrane segments that is equivalent to the separate protein CpsC in *S. pneumoniae*. A third transmembrane segment is located in the C-terminus of Wzc. The C-terminus also contains the ATP-binding and tyrosine kinase sites. In the CpsC-D system, CpsC is required for phosphorylation of CpsD, while the same is not true for the Wzc-Wzb system of *E. coli*. The Wzc component is capable of two-step phosphorylation events (intra-molecular and inter-molecular [27]). In addition, the *E. coli *Wzb component belongs to the low molecular weight protein tyrosine phosphatases, as it possesses the major signature motif CX_5_R(S/T) [28]. Thus, these two types of systems appear to have evolved separately to carry out the control of polymerization of extracellular polysaccharides that are assembled from repeating units. Examples of convergent evolution can be found among systems relying on phosphorylation for signal transduction, as has been shown for the PTP subgroups of phosphatases [9,12].

Similar phosphatase and kinase genes are present in the exopolysaccharide (EPS) biosynthesis locus of four EPS-producing strains of *Lactobacillus rhamnosus *(AY659976 [29]). The present study demonstrates the phosphatase activity of Wzb from *L. rhamnosus *strain ATCC 9595, its characteristics and the unique ion dependence profile of this enzyme. Site-directed mutagenesis is used to identify key residues involved in the catalytic activity.

## Results

### Comparative sequence analysis of Wzb

Among four strains of *L. rhamnosus*, Wzb is 100% identical, except for one amino acid change (T53 to A) for the Wzb of strain RW-6541M (GenBank Acc. No. AY659977) [29]. Wzb has the highest identity (94%) with CapC from *Lactobacillus casei *ATCC 334 (GenPept Acc. number YP_807246), and shows high similarity (74%; 39% identity) with PtpZ (YwqE; Swiss-Prot ID P96717) from *Bacillus subtilis *168, which dephosphorylates tyrosine residues [7,8]. Wzb shows low identity (24%) with the phosphotyrosine-protein phosphatase from *S. pneumoniae *strain D39 (Cps2B; GenBank Acc. No. AF026471), for which the phosphatase function has been demonstrated on the cognate tyrosine kinase Cps2D and on *p*-nitrophenyl phosphate [20].

**Figure 1:**
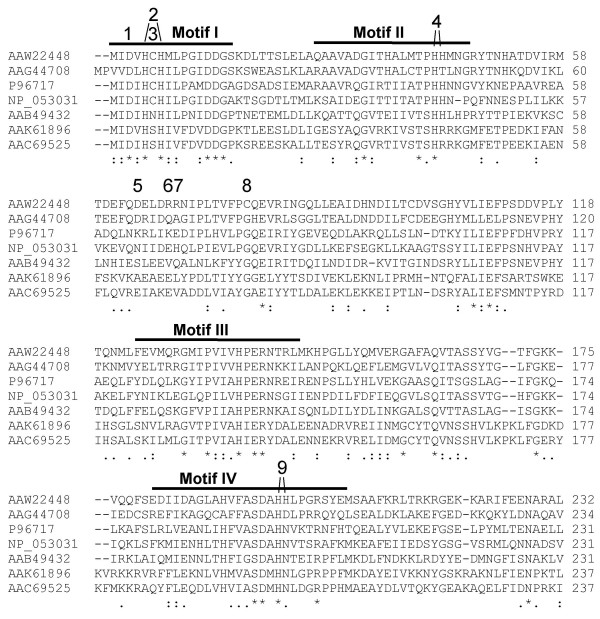
Sequence alignment of the predicted Wzb protein from *L. rhamnosus *strain ATCC 9595 (GenPept Acc. No. AAW22448). Similar proteins from polysaccharide biosynthesis loci are as follows: AAG44708 is from *Lactobacillus delbrueckii *subsp. *bulgaricus *Lfi5 (EpsD); P96717 is from *Bacillus subtilis *168 (YwqE/PtpZ); NP_053031 is from *Lactococcus lactis *subsp. *cremoris *B40 (EpsC); AAB49432 is from *Staphylococcus aureus *8C (Cap8C); AAK61896 is from *Streptococcus thermophilus *Sfi39 (EpsB); AAC69525 is from *Streptococcus pneumoniae *23F (Cps23fB). The four conserved motifs of the PHP domain (PF02811) are underlined, identical residues are indicated with an asterisk, while conserved residues and substitutions are indicated with dots. Mutations inserted in the *wzb *gene are numbered and give the following amino acid changes: 1: D3N, 2: H5A-H7A, 3: C6S, 4: H42A-H43A, 5: D64N, 6: R68A, 7: R69A, 8: C78S, 9: H197A-H198A (residue numbering corresponds to wild type Wzb amino acids).

Typical tyrosine and serine/threonine phosphatase consensus sequences are absent or modified in Wzb orthologs. In addition, the CX_5_R motif of PTP phosphatases is absent [2,30]. In fact, no cysteine residues are fully conserved in this alignment. Only the DXH is fully conserved (Fig. 1) out of the consensus sequence found in a wide variety of phosphoesterases (DXH(X)_n_-GDXXD(X)_m_GNHD/E [31,32]). This consensus sequence was also found in the multifunctional phosphoprotein phosphatase from lambda [31].

Phosphoesterase activity can be predicted for Wzb, based on alignment of deduced amino acid sequences with PHP superfamily members (PHP domain, PF02811). Four conserved motifs of the PHP (polymerase and histidinol phosphatase) domain identified by Aravind & Koonin [4] were located in the amino acid sequence of Wzb. These motifs consist of conserved histidine and aspartic acid residues (Fig. 1).

### Production, purification and phosphotyrosine activity of Wzb

**Figure 2:**
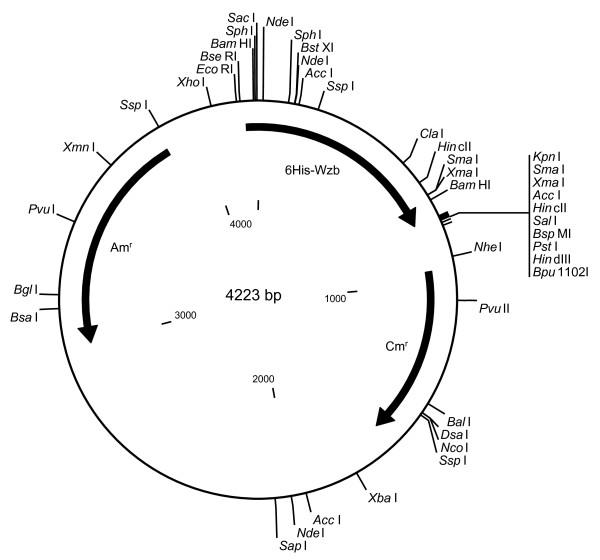
Physical map of pGL387 (pQE30 with the *wzb *ORF from *L. rhamnosus *strain ATCC 9595). 6H-Wzb = *wzb *gene cloned into *SacI/KpnI*; Am^r ^= ampicillin resistance; Cm^r ^= chloramphenicol resistance.

**Figure 3:**
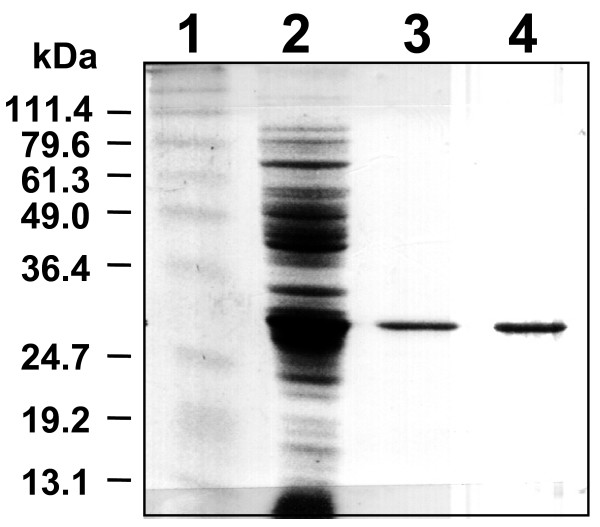
SDS-PAGE of His-tagged Wzb purification by affinity chromatography. Lane 1: Super Molecular marker (Bio-Rad, Mississauga, ON); Lane 2: *E. coli *NM522 (pGL387) IPTG-induced culture lysate; Lane 3: Fraction 1 from elution step; Lane 4: Fraction 2 from the elution step.

The gene coding for the putative O-phosphatase (*wzb*) from *L. rhamnosus *ATCC 9595 was expressed using the construct pGL387, producing a His-tagged fusion protein (Table 1; Fig. 2). *E. coli *(pGL387) cultures induced with IPTG showed over-production of a 27-kDa protein (Fig. 3) corresponding to the predicted His-tagged Wzb. Purified His-Wzb was eluted as a single band of the same molecular mass (Fig. 3). Phosphate release was shown by cell lysates in the presence of each of two phosphopeptides (Tyr-1 is END(pY)INASL and Tyr-2 is DADE(pY)LIPQQG). Induced cell extracts released 2.86 pmol PO_4 _min^-1 ^μg^-1 ^in the presence of Tyr-1, compared to 2.57 pmol PO_4 _min^-1 ^μg^-1 ^in the absence of the phosphopeptide (a difference of 587 pmol over the 45-min reaction time). When 1 mM sodium vanadate was added to the reaction, only 1.30 pmol PO_4 _min^-1 ^μg^-1 ^were released, representing a 55% reduction in activity.

**Table 1:** Bacterial strains, plasmids, and oligonucleotide primers.

Strain, plasmid, or primer	Relevant characteristic(s) or sequence (5' to 3')	Source, reference or target
**Strains**		
*L*. *rhamnosus *ATCC 9595	Low EPS-producing (116 mg/L)	ATCC^1^
*E*. *coli *XL1-Blue	Cloning host (*recA1 endA1 gyrA96 thi-1 hsdR17 supE44 relA1 lac *[F' *proAB lacI*^q^*Z*Δ*M15 *Tn*10*])	Stratagene^1^
*E*. *coli *NM522	Cloning host (*supE thi-1 *Δ(*lac-proAB*) Δ(*mcrB-hsdSM*)*5 *(rK- mK-) [F' *proAB lacI*^q^*Z*Δ*M15*])	Stratagene^1^
		
**Plasmids**		
pQE30	His-tag fusion protein expression vector; Cm^r^, Am^r^	Qiagen^1^
pGL387	768-pb digested PCR fragment (*wzb*) cloned into the *Sac*I-*Kpn*I site of pQE30	This study
		
**Primers**^2^		
BF1SacI	gagctcATTGATGTGCATTGCCATATGTTACCGGGA	PCR of *wzb*
BRSKpn	ggtaccTTAATACCGCGACAACAAACGCTTTTCAACC	PCR of *wzb*
BD4NF	ATTAATGTGCATTGCCATATGTTACCGGGA	*wzb*-D3N forward
BD4NR	TCCCGGTAACATATGGCAATGCACATTAAT	*wzb*-D3N reverse
BFHI	ATTGATGTGGCTTGCGCTATGTTACCGGGA	*wzb*-H5A-H7A forward
BRHI	TCCCGGTAACATAGCGCAAGCCACATCAAT	*wzb*-H5A-H7A reverse
BC6SF	ATTGATGTGCATAGCCATATGTTACCGGGA	*wzb*-C6S forward
BC6SR	TCCCGGTAACATATGGCTATGCACATCAAT	*wzb*-C6S reverse
BH42AF	TGATGACGCCGGCCGCTATGAATGGCCG	*wzb*-H42-H43A forward
BH42AR	CGGCCATTCATAGCGGCCGGCGTCATCA	*wzb*-H42-H43A reverse
BD64NF	GTTTCAAAACGAGTTAGACCGCCGCAATATTCCA	*wzb*-D64N forward
BD64NR	TGGAATATTGCGGCGGTCTAACTCGTTTTGAAAC	*wzb*-D64N reverse
BR68AF	GTTTCAAGACGAGTTAGACGCCCGCAATATTCCA	*wzb*-R68A forward
BR68AR	TGGAATATTGCGGGCGTCTAACTCGTCTTGAAAC	*wzb*-R68A reverse
BR69AF	GTTTCAAGACGAGTTAGACCGCGCCAATATTCCA	*wzb*-R69A forward
BR69AR	TGGAATATTGGCGCGGTCTAACTCGTCTTGAAAC	*wzb*-R69A reverse
BC78SF	TTTCCCGAGTCAGGAAGTGCGGATTAATGGGC	*wzb*-C78S forward
BC78SR	GCCCATTAATCCGCACTTCCTGACTCGGGAAA	*wzb*-C78S reverse
BFH197A	CCTCTGATGCCGCTGCCTTACCGGGTCGCAGTTA	*wzb*-H197-H198A forward
BRH197A	TAACTGCGACCCGGTAAGGCAGCGGCATCAGAGG	*wzb*-H197-H198A reverse

^1 ^ATCC (American Type Culture Collection, Manassas, VA, USA); Qiagen S.A. (Courtaboeuf, France); Stratagene (LaJolla, CA, USA).

^2 ^Restriction sites in primers are indicated in lower case letters.

### Optimal in vitro conditions for Wzb activity

**Figure 4:**
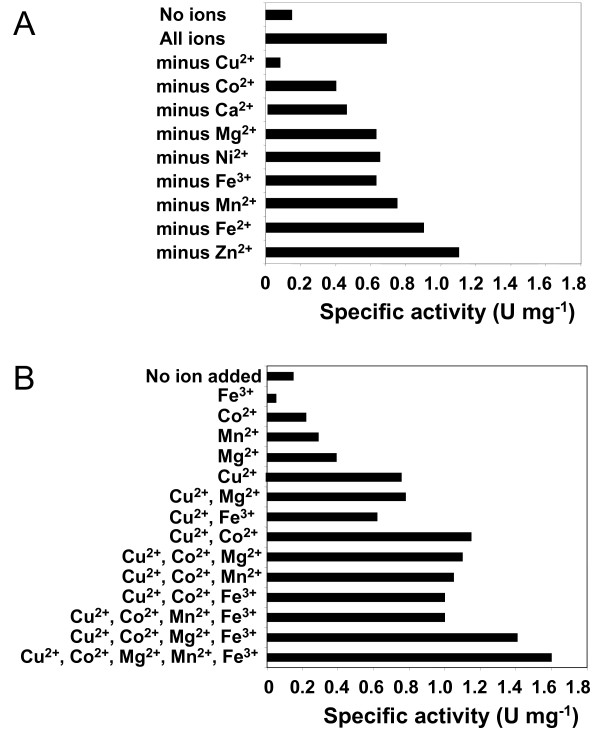
Effect of different ions on the specific activity of wild type Wzb. Reactions were carried out at 40°C with 2.5 mM pNPP as substrate in 50 mM MES – 50 mM HEPES, 1% BSA buffer, pH 8. Each ion was added at a final concentration of 0.1 mM. **A**. One ion was subtracted for each reaction type. **B**. Combinations of ions were added as indicated.

**Figure 5:**
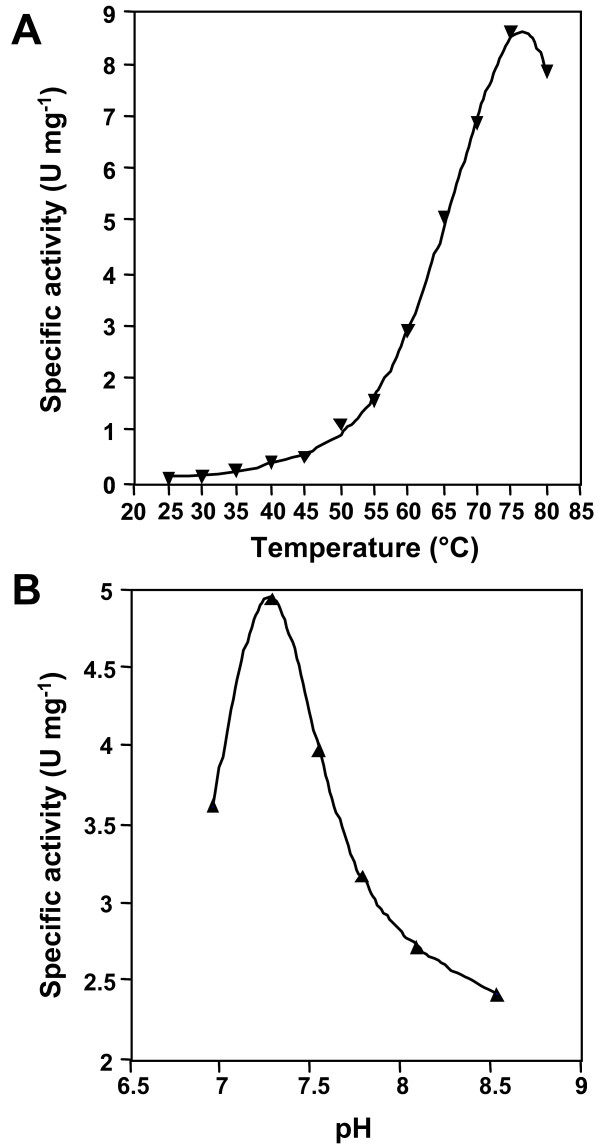
Effect of reaction conditions on the specific activity of wild type Wzb. **A**. Effect of temperature. Endpoint reactions were carried out in 50 mM MES – 50 mM HEPES buffer, pH 8, containing 1% BSA in the presence of Cu^2+^, Co^2+^, Fe^3+ ^(0.1 mM each) and Mn^2+^, Mg^2+ ^(0.5 mM each). A 30-min pre-incubation was carried out at the desired temperature before addition of 2.5 mM *p*NPP. Reactions were then stopped after 10 min incubation with 100 mM (final) sodium vanadate (Na_3_VO_4_). **B**. Effect of pH. Endpoint reactions were carried out in 100 mM MES – 100 mM HEPES buffer with 1% BSA in the presence of Cu^2+^, Co^2+^, Fe^3+ ^(0.1 mM each) and Mg^2+^, Mn^2+ ^(at 0.5 mM each). Incubation for 30 min at 75°C was carried out before the addition of 2.5 mM *p*NPP. Reactions were stopped after 10 min. with 100 mM (final concentration) sodium vanadate.

The purified Wzb fusion protein was active on *p*-nitrophenyl-phosphate (*p*NPP), and activity was most stable in the presence of 1% BSA. The presence of Cu^2+ ^ions (0.1 mM) was essential for activity, which was significantly increased by addition of Co^2+ ^(0.1 mM). Added individually, Mn^2+^, Mg^2+ ^and Fe^3+ ^did not have a significant effect, but, when added together, there is a significant increase in Wzb activity (Fig. 4). Optimal *p*NPP hydrolysis occurred in the presence of BSA (1%) at pH 7.3 (Fig. 5A) and a temperature of 75°C. Residual activity was only 10% at 50°C (Fig. 5B). Wzb activity on *p*NPP was inhibited by sodium orthovanadate (Na_3_VO_4_). Increasing concentrations from 1 mM up to 100 mM showed increasing inhibition of Wzb activity (data not shown). As no residual activity was found at 100 mM, this concentration of vanadate was used for stopping endpoint reactions.

### Effect of site-directed mutagenesis on Wzb phosphatase activity

**Figure 6:**
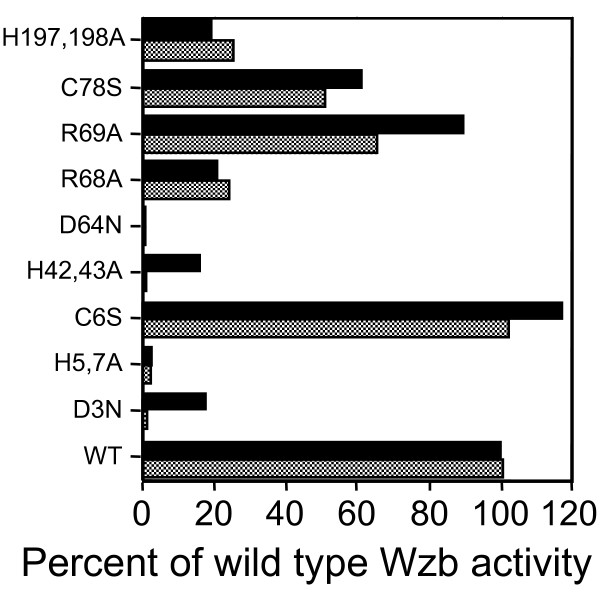
Relative activity of variant Wzb proteins. Endpoint reactions at 75°C (black bars) and kinetic reactions at 47°C (gray bars) were carried out in 50 mM MES, 50 mM HEPES buffer containing 1% BSA in the presence of Cu^2+^, Co^2+^, Fe^3+ ^(at 0.1 mM each) and Mn^2+^, Mg^2+ ^(at 0.5 mM each). In the case of endpoint reactions, a 30-min pre-incubation at 75°C was carried out before adding 2.5 mM *p*NPP. Reactions were stopped after 10 min. incubation by the addition of 100 mM (final conc.) sodium vanadate.

Residues targeted for site-directed mutagenesis were selected within the previously-identified PHP superfamily motifs (variants 1 to 4 and 9; Fig. 1), as well as aspartate, arginine and cysteine residues outside these motifs (variants 5, 6, 7, 8; Fig. 1). Replacement of histidine residues in positions 5 and 7, or 42 and 43 with alanine as well as replacement of aspartate residues in positions 3 or 64 with asparagine lead to drastic reductions of up to 99% of the specific activity of the mutated Wzb proteins (Fig. 6). Modification of the arginine residue in position 68 to alanine (R68A), or the histidines in position 197 and 198, also to alanine, leads to a 75% reduction in phosphatase activity. The reduction in activity was not as great when the arginine in position 69 (R69A; 10% to 30%) or the cysteine in position 78 (C78S; 40 to 50%) were changed. Finally, the substitution of the cysteine in position 6 by a serine (C6S) leads to an increase in phosphatase activity, more visibly when measured by the endpoint method than when measured by the kinetic method. In general, both the endpoint and kinetic reaction methods gave similar results, except for variants D3N, C6S, H42A-H43A and R69A, and to a lesser extent, the C78S variant, where the endpoint method resulted in higher specific activity than the kinetic method. This may be due to the lower temperature used in the kinetic reaction, which limits the activity that can be measured.

## Discussion

Phosphatases are involved in regulating many cell processes by reversing phosphorylation events. Their substrates may be as simple as inorganic pyrophosphate, or as complex as nucleotides, nucleic acids or proteins. Accumulating pyrophosphate must be hydrolysed in order to drive nucleotide polymerisation during DNA synthesis, while changes in the phosphorylation state of proteins may result in conformational changes, and thus changes in their function.

Such phosphorylation events are proposed to be responsible for the polymerisation of heteropolysaccharides. Repeating units are synthesized inside the cell through the successive action of glycosyltransferases using sugar nucleotides as substrates. The repeating units linked to a lipid carrier are then transferred to the cell surface where the polymers are assembled by a polymerization complex. Little is known of the structure or the mechanism of this polymerization complex. Inside the Gram positive bacterial cell, the proteins proposed to be involved in controlling the chain length of the polymers produced include a phosphatase, a tyrosine kinase and a co-polymerase membrane protein. Before any study of the mechanism can be carried out, demonstration of the function of the components and the characterization of their activity are necessary.

The biochemical characteristics of Wzb activity are unique and further define a new subfamily of bacterial PHP phosphatases that has not been well-characterized to date. Activity on phosphotyrosine peptides is equivalent to that demonstrated for CpsB of *S. pneumoniae *strain Rx1-19F on the same phosphopeptides [25]. However, dependence on copper and, to a lesser extent, on cobalt is original. Lambda phosphatase activity is not stimulated very much by cobalt and not at all by copper ions. In contrast, manganese and magnesium ions are essential for lambda phosphatase activity. This enzyme is a well-characterized PPP superfamily member that is multifunctional, showing activity on serine/threonine, tyrosine and histidyl phosphoproteins [20].

The optimal pH of 7.3 for Wzb activity suggests that as lactic acid accumulates during growth and both the external and internal pH of the cells decrease [33], Wzb phosphatase activity will also decrease. The optimal temperature of 75°C reflects the nature of the optimal in vitro biochemical reaction. However, at physiological temperatures of 30°C to 37°C, the phosphatase activity is controlled at a very low rate that should be consistent with the requirements of the cell.

The phosphatase orthologs found in many Gram positive exopolysaccharide biosynthesis loci belong to a distinct subfamily of the PHP superfamily of phosphoesterases with very diverse functions. Four motifs of histidine and aspartate residues are highly conserved among all members of this superfamily. Histidine residues are proposed to be involved in binding of divalent metal ions (such as Mn^2+^) in a catalytic site that is also coordinated by conserved aspartate or glutamate residues [3,4]. However, there are very few site-directed mutagenesis studies supporting this proposal. Study of the yeast histidinol phosphatase has demonstrated the requirement of some of these residues for phosphoesterase activity during histidine biosynthesis. Mutations of histidine residue 6 at the N-terminus of the conserved HXH of motif I, as well as of His390 in motif III result in a histidine-requiring phenotype, indicating that the enzymatic step is not functional [34]. In addition, inactivation of the aspartate or histidine residues of motif IV resulted in loss of activity for Cps2B from *S. pneumoniae *[25]. However, no study has yet demonstrated whether the remaining histidines were essential for phosphoesterase activity. The results presented here show that all conserved histidines, but especially those in positions 5 and 7, are required for optimal activity of the Wzb phosphatase. Mutation of histidines in positions 5 and 7 affect the secondary structure prediction for the variant protein, leading to an alpha helix replacing a coiled region, which could affect protein folding.

In fact, all nine of the mutated fusion Wzb proteins exhibited altered phosphatase activity on *p*-nitrophenyl-phosphate in comparison with the activity level of the wild-type Wzb fusion protein. Some phosphatases that are orthologs of Wzb contain a serine in position 6, not a cysteine, as does the Wzb of strain ATCC 9595. Changing this cysteine to serine (C6S) actually increases phosphatase activity slightly. In contrast, the activity of the C78S variant is decreased by 40%. Cysteine is involved in tyrosine-specific phosphatase activity for enzymes of the PTP superfamily [13]. However, the catalytic mechanism of Wzb does not appear to rely heavily on these cysteine residues.

In addition, certain other amino acids not part of the four histidine motifs are essential for Wzb activity. Mutations of D3 or D64 to asparagine are particularly critical, indicating the requirement for aspartic acid residues in coordinating the catalytic site. Residue D3 forms part of the general phosphoesterase consensus sequence (as found in the lambda phosphatase), but is not invariantly conserved in other members of the PHP superfamily. Residue D64 is located outside of the four general PHP motifs, although it is part of a DXXDR motif also found in the lambda phosphatase consensus sequence. For the lambda phosphatase, aspartate residues, along with histidine residues, were shown to contribute to both metal binding and catalysis, as protein variants D20N, H22N, D49N and H76N showed decreased *p*NPP hydrolysis rates as well as reduced affinity for Mn^2+ ^[31].

Mutation of two arginine residues also reduced Wzb phosphatase activity, although not to an equivalent extent (80% reduction for R68A in comparison to 20–40% reduction for R69A). Arginine residues are an essential part of catalysis and substrate binding for the lambda phosphatase [31] as well as for phosphatases of the PTP superfamily [2], and are involved in binding the oxygen atoms of the phosphoryl group [35]. Mutations R53A and R73A in lambda phosphatase resulted in reduced affinity for *p*NPP and phosphate binding, but metal ion affinity was not affected [31]. The data from our study are consistent with these results, as enzyme activity was reduced, but not completely inhibited, for Wzb variants R68A and R69A. Mutation of arginine residues may affect substrate recognition, possibly through differences in protein interactions or folding. The fact that these two arginine residues are unique to the Wzb from *L. rhamnosus *strains may indicate different substrate specificity for this enzyme. It is easily conceivable that phosphatases involved in EPS polymerization complexes recognize a specific cognate phosphotyrosine protein, as suggested by Bender and Yother [20] as well as Morona et al. [25]. Protein tyrosine kinases of lactic acid bacteria vary in the positioning of the series of tyrosine residues that are phosphorylated, thus suggesting that the phosphatase substrate-recognition sequence should vary accordingly. There are a total of 19 arginine residues (7.5%) in the sequence of Wzb, of which only two are completely conserved among the phosphatase proteins aligned. Interestingly, these two arginine residues are positioned close to histidines in motifs III and IV. This situation is not unique to Wzb, as the Cps23fB phosphatase has 17 arginines (7.0%) in its sequence.

Caution must thus be exerted in the interpretation of the exact nature of the predicted phosphoesterase activity, as more experimental analysis necessary in order to reveal other potential activities of PHP phosphatases. Multiple independent fusions of phosphoesterase domains with polymerases indicate a selective advantage to this association. A unifying explanation for this phenomenon is shifting the reaction equilibrium towards polymerization. Some PHP phosphoesterases may thus accomplish an allosteric regulatory function by binding pyrophosphate that accumulates during DNA polymerization [4]. In an analogous fashion, the polysaccharide polymerization complex may require the binding and hydrolysis of pyrophosphate in order to favor the shift towards polymerization. The pyrophosphate could be produced from the regeneration of the lipid carrier, released after polymerization of each repeating unit. This would explain why such a polyvalent enzyme would be associated with this phosphorylation system. Therefore, it cannot be excluded that the phosphatase activity of Wzb could play a role in EPS polymerisation, independently of the cognate protein tyrosine kinase, while also able to dephosphorylate this protein.

## Conclusion

The distinct properties of Wzb from *L. rhamnosus *ATCC 9595 underscore the diversity in function of the members of various subfamilies of PHP phosphoesterases. The essential character of certain amino acids and a dependence on the presence of copper should contribute to identifying the active site. Future perspectives include interactions of the phosphatase with other proteins potentially involved in the polymerization complex, in order to determine their role in modulating the biosynthesis of exopolysaccharides on the surface of *L. rhamnosus *cells.

## Methods

### Bacterial strains and culture conditions

*L. rhamnosus *strain ATCC 9595 was grown without agitation at 37°C in MRS medium [36]. *Escherichia coli *strains NM522 and XL1-Blue were grown at 37°C in low salt Luria Bertani (LB) medium with agitation. For *E. coli *transformants, ampicillin was added at 100 μg ml^-1^. Media were solidified when necessary with 1.5% Bacto Agar (Difco). All bacterial strains were maintained at -80°C in 20% glycerol.

### DNA manipulations

Genomic DNA was extracted as follows. Cells from 16–18 h cultures (2 ml) were harvested by centrifugation (8000 × g, 10 min) and washed with 1 ml of 200 mM sodium acetate solution. The pellet was suspended in 400 μl of 100 mM Tris-HCl pH 7.0, 10 mM EDTA, 25% glucose and incubated at 37°C for 2 h with 20 μl of 2 mg ml^-1 ^mutanolysin solution. After centrifugation (15000 × g, 10 min), cells were suspended in 400 μl of 100 mM Tris-HCl pH 7.0, 10 mM EDTA, 0.5% sarcosyl and incubated with proteinase K (400 μg) and RNAse A (500 μg) for 1 h at 37°C. Two extractions with phenol/chloroform and one with chloroform were carried out, followed by DNA precipitation with 95% ethanol and 3 M potassium acetate (pH 4.8). The pellet was washed twice with 70% ethanol and solubilized in 100 μl of 10 mM Tris-HCl pH 8.0.

PCR was performed using standard conditions [37] with *Taq *Polymerase (Promega, Madison, WI) and the primers listed in Table 1 specific for the *wzb *sequence from *L. rhamnosus *strain ATCC 9595 (GenBank Acc. No. AY659976) [29]. The forward primer was BF1Sac and the reverse primer was BRSKpn. The amplicon was then digested with *Sac*I and *Kpn*I, followed by ligation to *Sac*I-*Kpn*I digested pQE30 (Qiagen) and *E. coli *strain NM522 was transformed with the resulting construct, named pGL387.

Site directed lesions in pGL387 were inserted using the QuikChange^® ^kit (Stratagene, LaJolla CA) based on the work of Fisher and Pei [38]. Plasmid pGL387 was used a template (20 ng) in amplification reactions of 50 μl containing 1× *Pfu *DNA polymerase buffer, 25 μM each primer, 10 mM dNTP mix and 2.5 U *Pfu *DNA polymerase. PCR conditions consisted of 30 s at 95°C followed by 18 cycles of 95°C for 30 s, 55°C for 1 min, 68°C for 1 min. Methylated (parental) DNA was then degraded with *Dpn*I by adding 1 μl *Dpn*I (10 U) to each PCR reaction and incubating at 37°C for 1 h, followed by transformation of *E. coli *strain XL1-Blue. After transformant screening, selected recombinant plasmids were extracted by the alkaline lysis method and *E. coli *strain NM522 was transformed for production of fusion proteins.

The inserts of selected constructs were sequenced on both strands using universal primers by Cogenics (Meylan, France) with plasmid DNA as target. DNA and protein sequence analysis and similarity searches were carried out using the BLAST network service at the National Center for Biotechnology Information, National Institutes of Health, Bethesda, Md. [39]. Protein secondary structure was predicted using HNN (Hierarchical Neural Network method) at the NPS@ web server (Network Protein Sequence Analysis [40]).

#### Production and purification of Wzb fusion proteins

Small-scale volumes (1.5 ml) of transformants inoculated from an overnight culture (LB with 100 μg ml^-1 ^ampicillin) were induced with 0.1 M IPTG for 3 h, then the bacterial pellet was suspended in lysis buffer (0.1 M Tris-HCl, pH 6.8, 2 % SDS, 20 % glycerol, 0.005 % bromophenol blue and 0.1 volume of β-mercaptoethanol) in order to screen by SDS-PAGE for overproduction of the fusion protein.

For large scale purification, an overnight culture was used to inoculate (1% v/v) 400 ml of LB supplemented with ampicillin (100 μg ml^-1^), which was incubated at 37°C with shaking until A_600 _reached 0.6. Induction was initiated by adding IPTG to 1 mM and incubation continued for 3 h with shaking at 37°C.

Cells were harvested and lysed in 15 ml buffer A (25 mM Tris, pH 8.0, 500 mM NaCl, 6 M urea) containing 1 mg ml^-1 ^lysozyme. The resulting suspension was centrifuged and the supernatant was added to Ni^2+^-NTA-agarose resin. Batch binding was carried out for 1 h with gentle stirring. The lysate/resin mixture was loaded into a polypropylene column and protein renaturation was carried out on the column by applying a gradient of decreasing urea concentration over 90 min using Buffer B (25 mM Tris, pH 8.0, 300 mM NaCl, 20 mM imidazole, 20% glycerol). After washing with buffer B, proteins were eluted with buffer C (25 mM Tris, pH 8.0, 300 mM NaCl, 250 mM imidazole, 20% glycerol) and fractions were analyzed by SDS-PAGE. Fractions containing purified 6His-tagged proteins were pooled and stored at -20°C.

### Assays of Wzb phosphatase activity

Release of phosphate from tyrosine phosphopeptides was quantified using the Tyrosine Phosphatase Assay System (Promega France). Extracts were pretreated to remove endogenous phosphate according to the instructions of the manufacturer, then assayed by incubation for 45 min at 37°C in the presence or absence of each of two synthetic phosphopeptides (Tyr Phosphopeptide-1: END(pY)INASL or Tyr Phosphopeptide-2: DADE(pY)LIPQQG). After adding the molybdate dye solution, phosphate released from 50 μl of extract containing 45 μg protein was calculated from absorbance measurements (*A*_600_) using a calibration curve determined with known concentrations of free phosphate.

The effect of combinations of ions on kinetic reactions was conducted at 40°C using 2.5 mM *p*NPP in 50 mM MES- 50 mM HEPES buffer, pH 8, containing 1% BSA and with or without the presence of 0.1 mM each of the following ions: Ca^2+^, Co^2+^, Cu^2+^, Fe^3+^, Fe^2+^, Mn^2+^, Mg^2+^, Ni^2+^and Zn^2+^. The effect of temperature was measured using endpoint reactions in 50 mM MES – 50 mM HEPES buffer, pH 8, containing 1% BSA in the presence of Cu^2+^, Co^2+^, Fe^3+ ^(0.1 mM each) and Mn^2+^, Mg^2+ ^(0.5 mM each). Endpoint reactions showing the effect of pH were carried out in 100 mM MES, 100 mM HEPES buffer with 1% BSA in the presence of Cu^2+^, Co^2+^, Fe^3+ ^(0.1 mM each) and Mg^2+^, Mn^2+ ^(at 0.5 mM each). Reactions were pre-incubated for 30 min at 75°C before the addition of 2.5 mM *p*NPP (final conc.), incubated for 10 minutes, and then stopped by adding sodium vanadate to a final concentration of 100 mM. All samples were measured against a control (same composition and treatment as sample except that the sample aliquot was replaced with buffer C) using a double beam spectrophotometer. Assays of phosphatase activity of site-directed mutant proteins were carried out by both endpoint (at the optimal temp. of 75°C) and kinetic reactions (at 47°C in order to have sufficient activity within the temperature constraints of the spectrometer). Cleavage of *p*NPP was monitored by increase in absorbance at 405 nm and activity calculated using the molar extinction coefficient of 18,000 M^-1 ^cm^-1^. One unit is defined as 1 mmol of pNPP hydrolyzed min^-1 ^[41].

## Abbreviations

**EPS **= exopolysaccharide, ***p*NPP **= *para*-nitrophenyl-phosphate, **PHP **= polymerase and histidinol phosphatase, **PPP **= phosphoprotein phosphatase, **PPM **= magnesium or manganese-dependent phosphatase.

## Authors' contributions

GL conceived of the study with the participation of DA and CG in experimental design. GL carried out the alignments, constructed the mutants, demonstrated the phosphatase activity as well as drafted the manuscript. CG carried out the enzyme assays and kinetic analyses. DA and CG participated in revising the manuscript. All authors confirm that they have read and approved the final manuscript.
